# New Species, New Records, and Updated Key to the *Paravelia* (Hemiptera: Heteroptera: Veliidae) from Brazil †

**DOI:** 10.3390/insects13060541

**Published:** 2022-06-11

**Authors:** Juliana Mourão dos Santos Rodrigues, Felipe Ferraz Figueiredo Moreira

**Affiliations:** Laboratório de Biodiversidade Entomológica, Instituto Oswaldo Cruz, Fundação Oswaldo Cruz, Avenida Brasil, 4365, Pavilhão Mourisco, Sala 214, Manguinhos, Rio de Janeiro 21040-360, Brazil; ppmeiameiameia@gmail.com

**Keywords:** aquatic insects, Caatinga, geographic distribution, Gerromorpha, Neotropical region, taxonomy, water striders

## Abstract

**Simple Summary:**

True bugs (Hemiptera: Heteroptera) of the infraorder Gerromorpha are known as semiaquatic bugs due to their ability to walk on the surface of water. Veliidae is the most diverse family of semiaquatic bugs, including six subfamilies, 61 genera, and more than 960 described species. The genus *Paravelia* Breddin, 1898 (Veliidae: Veliinae), or broad-shouldered water-striders, occurs in a wide variety of lentic and lotic freshwater habitats, and is represented in Brazil by 38 species. Although the diversity of *Paravelia* in the country is relatively well known, eight states remain with no records of the genus. Aiming to fill these gaps, several expeditions were carried out between 2018 and 2021. As a result, a new species is described from Pernambuco State, three species are recorded for the first time in Ceará, Pernambuco and Piauí states, and an updated key to the Brazilian species of the genus is provided.

**Abstract:**

The broad-shouldered water-strider genus *Paravelia* Breddin, 1898 (Hemiptera: Heteroptera: Veliidae: Veliinae) is currently represented in Brazil by 38 species. Although the diversity of the genus in the country is relatively well known, eight states remain with no records of any species: Acre, Alagoas, Ceará (CE), Paraíba, Paraná, Pernambuco (PE), Piauí (PI), and Tocantins. Aiming to fill these gaps, several expeditions were carried out at conservation areas of the Caatinga biome between 2018 and 2021: Aiuaba Ecological Station (CE), Catimbau National Park (PE), and Serra das Confusões National Park (PI). *Paravelia luisi* sp. nov., a new species from PE, is described and illustrated. In addition, new records of *P. bilobata* Rodrigues, Moreira, Nieser, Chen & Melo, 2014, *P. digitata* Rodrigues & Moreira, 2016, and *P. nieseri* Moreira & Barbosa, 2012, and an updated key to the Brazilian species of *Paravelia* are provided. This study increases the number of species of *Paravelia* known in Brazil to 39 and provides the first records of the genus from three states: PE, with two species, and CE and PI, with one species each.

## 1. Introduction

The subfamily Veliinae (Hemiptera: Heteroptera: Veliidae) is currently represented in Brazil by six genera and 62 species: *Callivelia* Polhemus, 2021 (3 species), *Oiovelia* Drake & Maldonado-Capriles, 1952 (8 species), *Paravelia* Breddin, 1898 (38 species), *Platyvelia* Polhemus & Polhemus, 1993 (1 species), *Steinovelia* Polhemus & Polhemus, 1993 (2 species), and *Stridulivelia* Hungerford, 1929 (10 species) [1,2,3,4,5,6,7,8,9].

The broad-shouldered water-striders of the genus *Paravelia* can be recognized mainly by the absence of lateral tubercles on the mesoacetabulum and metasternum, the tarsomere II of the middle leg usually 4 to 5 times longer than the tarsomere I, the pretarsus of the middle and hind legs with setae-shaped arolia and two falcate claws, and the macropterous form usually with two maculae on each forewing [4].

Although *Paravelia* includes more than half of the known species of Veliinae in Brazil, eight of the 26 states of the country have no records of the genus (30.8%; Acre, Alagoas, Ceará, Paraíba, Paraná, Pernambuco, Piauí, and Tocantins), six have only one species recorded (23.1%; Amapá, Mato Grosso do Sul, Rio Grande do Norte, Rio Grande do Sul, Roraima, and Sergipe), and five have two species recorded (19.2%; Bahia, Goiás, Rio de Janeiro, Rondônia, and Santa Catarina). The remaining seven states (26.9%) have the highest concentration of known species: Pará (12), Amazonas (10), Mato Grosso (9), Minas Gerais (7), São Paulo (7), Espírito Santo (5), and Maranhão (4) [10].

In this study, we present the description of a new species of *Paravelia* from Pernambuco state, northeastern Brazil, and provide new records of *P. bilobata* Rodrigues, Moreira, Nieser, Chen & Melo, 2014, *P. digitata* Rodrigues & Moreira, 2016, and *P. nieseri* Moreira & Barbosa, 2012, as well as an updated key to the Brazilian species of this genus.

## 2. Materials and Methods

The material examined was collected during expeditions carried out between 2018 and 2021 at Aiuaba Ecological Station (Ceará state), Catimbau National Park (Pernambuco state), and Serra das Confusões National Park (Piauí state), as part of the project “Diversidade e Conservação de Hemiptera (Insecta) da Caatinga”. The collections were conducted with permits from the National System of Biodiversity Authorization and Information (SISBIO; permits #62159-1, #62159-2, #62159-3, #62159-4 and #62159-5). Specimens are deposited in the Coleção Entomológica do Instituto Oswaldo Cruz, Fundação Oswaldo Cruz, Rio de Janeiro, Brazil (CEIOC) and in the Coleção Zoológica do Maranhão, Universidade Estadual do Maranhão, Caxias, Brazil (CZMA). Terminology, description, and measurements follow the standards established in the latest revision of the genus [1].

All measurements are given in millimeters. Abbreviations used for measurements are as follows: body length (BL), head length (HL), head width through eyes (HW), length of antennomeres I–IV [without intersegmental pieces] (ANT I, ANT II, ANT III, ANT IV), maximum eye width (EYE), pronotum length on midline (PL), pronotum width (PW), length of foreleg segments (FORELEG), length of midleg segments (MIDLEG), length of hindleg segments (HINDLEG), femoral length (FEM), tibial length (TIB), length of tarsomeres I–III (TAR I, TAR II, TAR III).

Photographs of the specimens were obtained using a Leica M205 C stereomicroscope coupled with a digital camera and captured using the Leica LAS imaging system. Geographic coordinates of the collecting localities were obtained with a GPS receiver. Maps were produced using ArcGIS v. 10.5 (ESRI Inc., Redlands, CA, USA). In the key, we present the distribution of each species in the Brazilian states according to Moreira [10]. Abbreviations of the states’ names are according to the official standard IBGE [11].

## 3. Results

### 3.1. New Species

**Figure 1:**
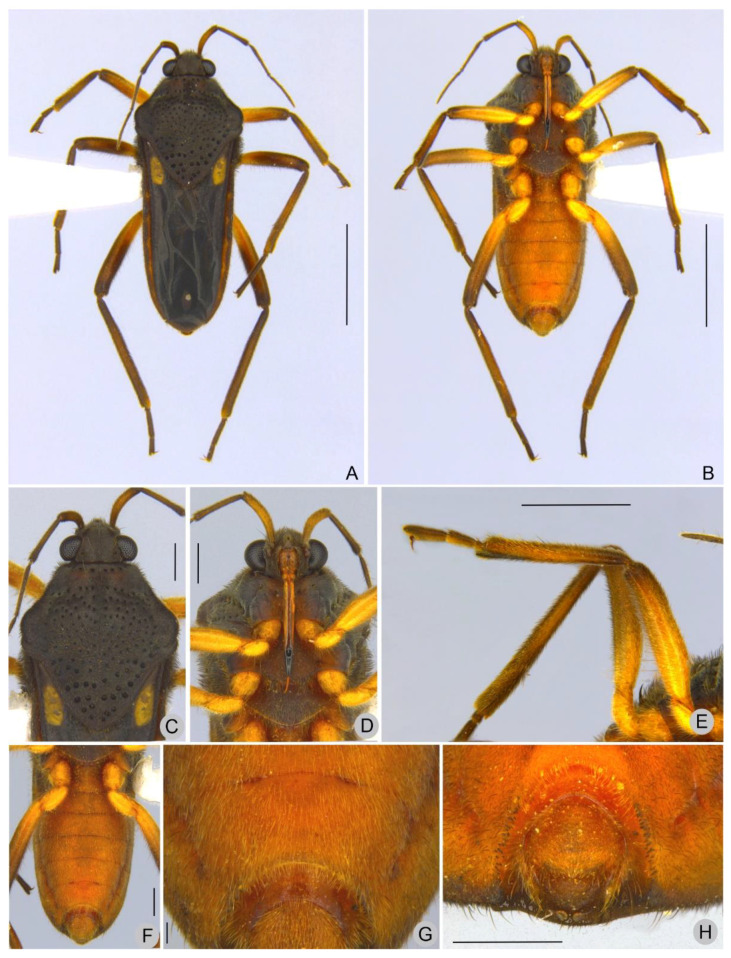
*Paravelia luisi* sp. nov., holotype male. (**A**). Habitus, dorsal view. (**B**). Habitus, ventral view. (**C**). Head, pronotum, part of the antennae and of the forewings, dorsal view. (**D**). Head, thoracic sterna, acetabulae, coxae and trochanters, ventral view. (**E**). Fore and midlegs, ventral view. (**F**). Abdomen and part of the hindlegs, ventral view. (**G**). Abdominal sterna VI [part], VII, and VIII [part], ventral view. (**H**). Apex of abdomen, posterior view. Scale bars: 2.0 mm (**A**,**B**), 1.0 mm (**E**), 0.5 mm (**C**,**D**,**F**,**H**), 0.1 mm (**G**).

**Figure 2:**
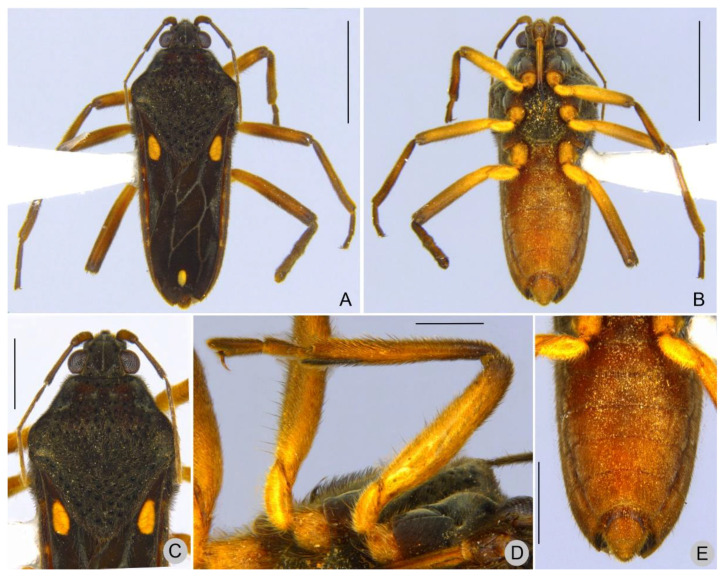
*Paravelia luisi* sp. nov., paratype male. (**A**). Habitus, dorsal view. (**B**). Habitus, ventral view. (**C**). Head, pronotum, part of the antennae and of the forewings, dorsal view. (**D**). Fore and mid legs, ventral view. (**E**). Abdominal sterna, ventral view. Scale bars: 2.0 mm (**A**,**B**), 1.0 mm (**C**,**E**), 0.5 mm (**D**).

**Figure 3:**
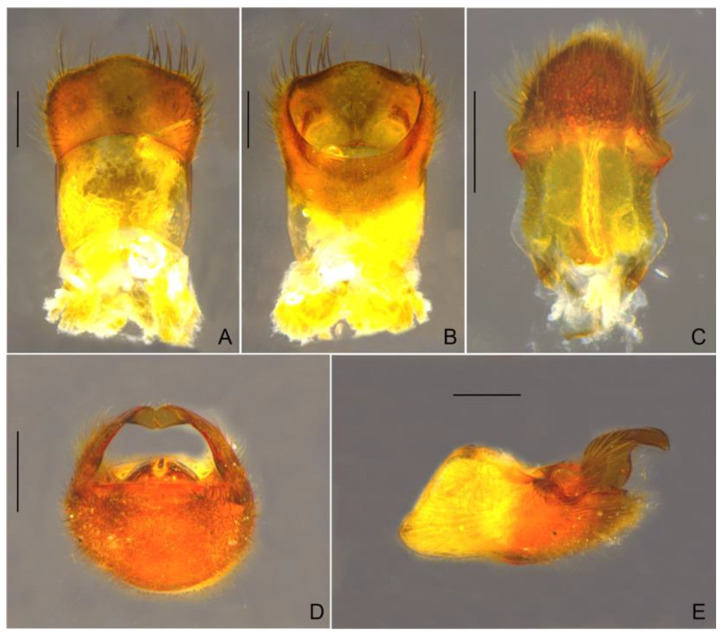
*Paravelia luisi* sp. nov., male terminalia. (**A**,**B**). Abdominal segment VIII. (**A**). Dorsal view. (**B**). Ventral view. (**C**). Proctiger, dorsal view. (**D**,**E**). Genital capsule. (**D**). Anterior view. (**E**). Lateral view. Scale bars: 0.2 mm.

**Figure 4:**
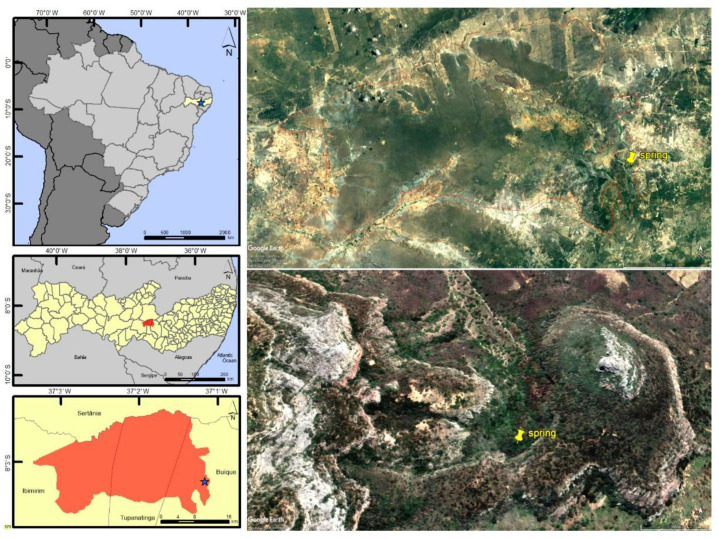
Geographic distribution of *Paravelia luisi* sp. nov. in the Catimbau National Park, Pernambuco state, Brazil. Right side images were produced using Google Earth.

**Figure 5:**
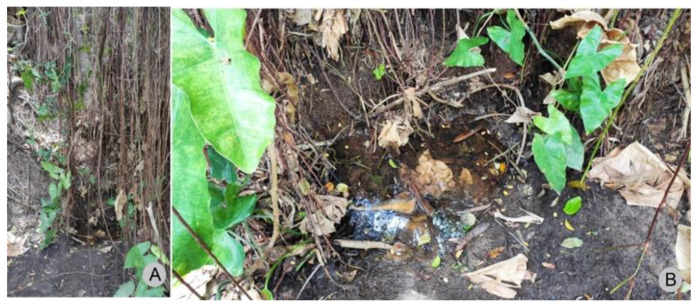
Photographs of the spring where *Paravelia luisi* sp. nov. was collected. (**A**). Distant view, showing the environment around the spring. (**B**). Close up view.

***Paravelia luisi,* sp. nov.** (Figure 1, Figure 2, Figure 3, Figure 4 and Figure 5)

urn:lsid:zoobank.org:act:70BB17D2-AA4C-47AA-AF9A-1AEED7C6FC31

Type-material. Holotype. BRAZIL–Pernambuco • Buíque, Parque Nacional do Catimbau, Sítio Arqueológico Alcobaça, nascente; alt. 755 m; 08°32′29.2″ S, 37°11′41.6″ W; 14.III.2021; J.M.S. Rodrigues & R. Jordão leg.; 1 macropterous ♂, CZMA. Paratypes. BRAZIL–Pernambuco • same data as holotype; 5 macropterous ♂ CEIOC.

Description. Macropterous males (Figure 1, Figure 2 and Figure 3). Holotype/paratypes. BL 5.55/5.30–5.60; HL 0.50/0.50–0.62; HW 1.02/1.00–1.02; ANT I 0.72/0.67–0.75; ANT II 0.65/0.60–0.65; ANT III 0.70/0.60–0.72; ANT IV 0.77/0.70–0.75; EYE 0.27/0.27; PL 2.25/2.22–2.30; PW 2.10/2.05–2.15; FORELEG, FEM 1.42/1.40–1.50; TIB 1.30/1.22–1.40; TAR I 0.07/0.07; TAR II 0.25/0.22–0.25; TAR III 0.40/0.37–0.42; MIDLEG, FEM 1.75/1.65–1.80; TIB 1.75/1.57–1.85; TAR I 0.06/0.06; TAR II 0.46/0.45–0.57; TAR III 0.46/0.45–0.52; HINDLEG, FEM 2.10/2.05–2.15; TIB 2.32/2.20–2.40; TAR I 0.07/0.07; TAR II 0.52/0.50–0.57; TAR III 0.45/0.47.

General color dark-brown to black. Head dark-brown. Antenna dark-brown; antennomere I yellowish-brown with base and apex dark-brown. Labium with two basal segments brown, segment III yellow laterally and brown medially, distal segment blackish. Pronotum dark-brown with two horizontal brown maculae anteriorly. Thoracic sterna dark-brown, lighter centrally. Acetabulae dark-brown laterally, yellowish-brown mesally. Forewing dark-brown with basal ovate yellow macula not reaching humeral angle and ending before posterior margin of pronotum, at apex another rounded to oval yellowish-white to yellow macula, smaller than basal one (Figure 1A,C and Figure 2A,C); veins whitish. Abdominal laterotergites light-brown mesally, dark-brown laterally; intersegmental area with a yellowish macula. Coxae, trochanters, femora, tibiae and tarsi yellow; femora and tibiae dorsally dark-brown. Abdomen and terminalia light brown.

Head covered by fine golden pubescence intermixed with elongate dark-brown setae; dorsal midline impressed, shiny, posteriorly with pair of oblique, impressed, shiny lines and pair of indentations near mesal margins of eyes; buccula and jugum without black denticles. Ocular setae present. Antenniferous tubercle developed, shiny. Antenna covered by golden pubescence and elongate golden setae scattered on segments II–IV; antennomere I more robust, curved laterally; II thicker than III–IV.

Pronotum covered by fine golden pubescence, intermixed with elongate dark-brown setae; anterior lobe with row of rounded punctations adjacent to anterior margin; posterior lobe covered by rounded punctations, these larger towards apex; humeral angle slightly elevated; posterior angle slightly tapered, apex rounded. Forewing reaching tip of abdomen, leaving only posterior portion of terminalia exposed; with four closed cells; veins on basal half with elongate dark-brown setae. Proepimeron with rounded punctations. Meso- and metapleura with scattered rounded punctations. Prosternum with row of four rounded punctations anteriorly. Meso- and metasterna centrally with two pairs of small tubercles on intersegmental region. Legs densely covered with short, appressed, pale setae and elongate, brownish setae. Fore tibia with grasping comb (0.50 mm long) occupying 1/3 of its length. Hind femur without spines.

Abdominal laterotergites covered by golden pubescence; elevated, without black denticles. Abdominal sterna covered by fine golden pubescence and elongate dark brown setae, the latter concentrated on lateral margins; II compressed laterally and bearing weak longitudinal carina anteriorly; VII without projections or nodules, with posterior margin evenly concave and with robust black denticles (Figure 1H). Abdominal segment VIII (=first genital segment in older literature) with fine golden pubescence on apical 2/3 intermixed with elongate dark-brown setae dorsolaterally; with black denticles on lateral areas (Figure 3A). Proctiger with elongate golden setae on apical half, without spines, with a pair of lateral projections in dorsal view (Figure 3C). Paramere, in lateral view, anteriorly notched on dorsal surface, sinuous, with elongate golden setae and rounded apex (Figure 3E).

Female. Unknown.

Intraspecific variation. Two paratypes collected in the same locality as the holotype have the apical macula of the forewing distinctly yellow and larger than in other specimens (Figure 2A).

Etymology. The new species is named in honor of Luis Cavalcanti Ramos, an exceptional field guide who accompanied us during the expedition and indicated the place where we found the specimens.

Distribution and habitat (Figure 4 and Figure 5). *Paravelia luisi* sp. nov. was collected in a spring located in the Catimbau National Park, a conservation area of the Caatinga biome. This is the first record of the genus Paravelia from Pernambuco state, northeastern Brazil.

Comments. This new species differs from the others of the genus by the body length (5.30–5.60 mm); the dark-brown coloration contrasting with the light-brown color of the abdomen and legs; the anterior lobe of the pronotum without marked pruinosity or pubescence, with two horizontal brown maculae anteriorly; the posterior angle of the pronotum slightly tapered and the apex rounded; the maculae of the forewing yellowish-white to yellow, consisting of: a basal ovate macula not reaching the humeral angle and ending before the posterior margin of the pronotum, and an apical oval macula, smaller than the basal one; the absence of spines or teeth on the legs; the absence of rounded punctures on abdominal sterna; the absence of small black denticles on the body, except for those located on the apex of male abdominal segment VII and dorsolaterally on male abdominal segment VIII; and the male without lobes or projections on abdominal sternum VII.

### 3.2. New Records

**Figure 6:**
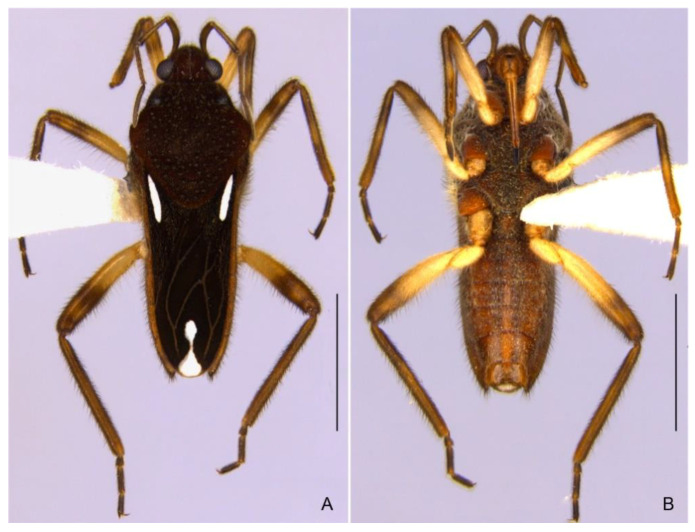
*Paravelia bilobata*, macropterous male from Aiuaba, Ceará state. (**A**). Dorsal view. (**B**). Ventral view. Scale bars: 2.0 mm.

**Figure 7:**
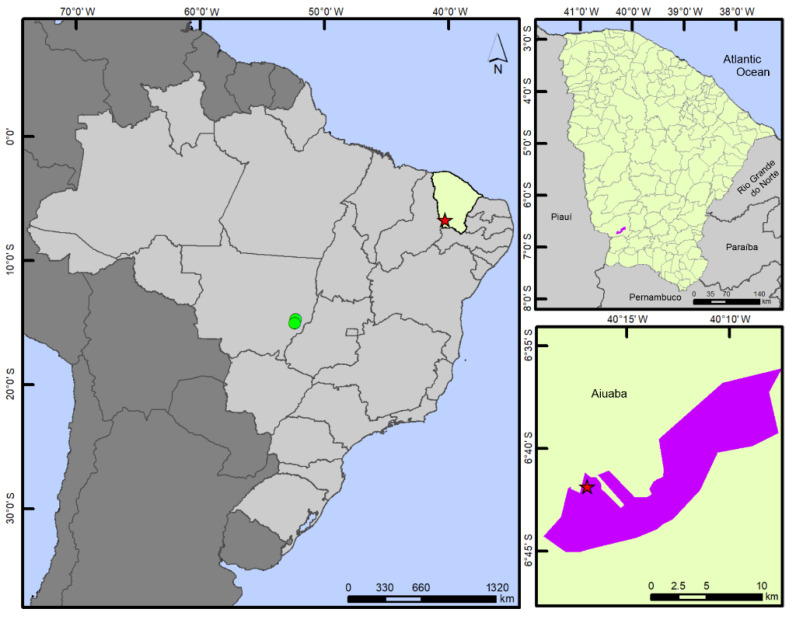
Geographic distribution of *P. bilobata*. Green circles show the previous records in Mato Grosso state. Red star shows the new record from Aiuaba Ecological Station (highlighted in purple), Ceará state (highlighted in green).

***Paravelia bilobata* Rodrigues, Moreira, Nieser, Chen & Melo, 2014** (Figure 6 and Figure 7)

Material examined. BRAZIL–Ceará • Aiuaba, Estação Ecológica de Aiuaba, estrada; alt. 505 m; 06°41′50.7″ S, 40°16′54.8″ W; 08.VI.2021; light trap; J.M.S. Rodrigues leg.; 1 ♂, CEIOC.

Distribution. Brazil (CE*, MT).

**Figure 8:**
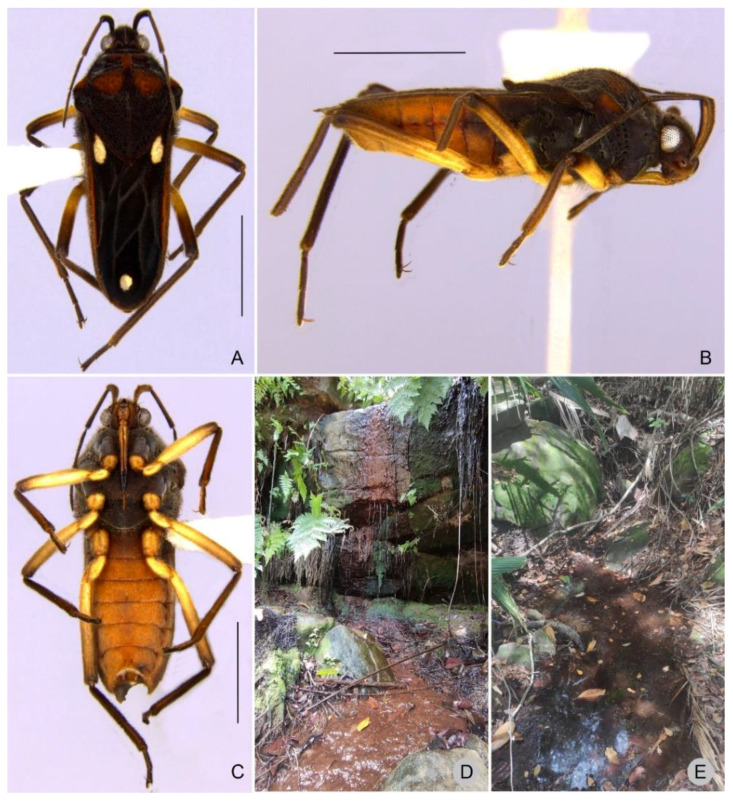
*Paravelia digitata* from Piauí state. (**A**–**C**). Habitus. (**A**). Macropterous female, dorsal view. (**B**,**C**). Macropterous male. (**B**). Lateral view. (**C**). Ventral view. (**D**,**E**). Photographs of the stream where the species was collected in Catimbau National Park (08°33′27.6″ S, 37°11′42.2″ W; 15.III.2021). Scale bars: 2.0 mm.

**Figure 9:**
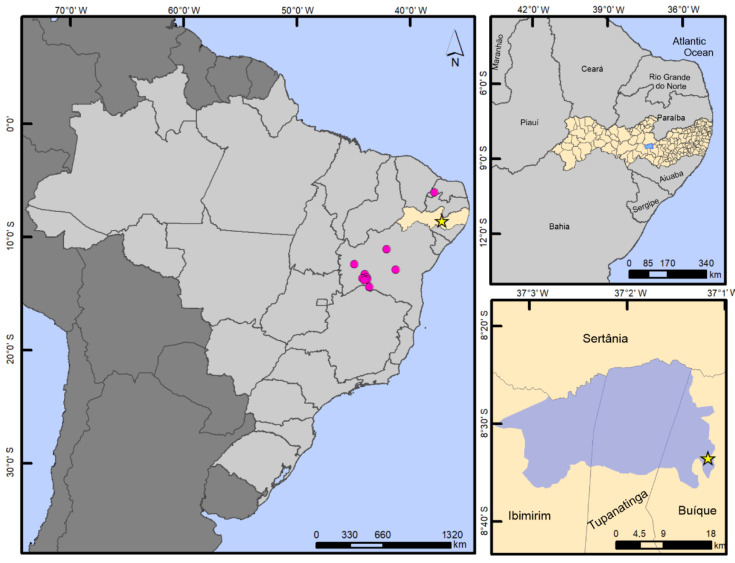
Geographic distribution of *P. digitata*. Pink circles show the previous records in Rio Grande do Norte and Bahia states. Yellow star shows the new record from Catimbau National Park (highlighted in blue), Pernambuco state (highlighted in beige).

***Paravelia digitata*Rodrigues & Moreira, 2016** (Figure 8 and Figure 9)

Material examined. BRAZIL–Pernambuco • Buíque, Parque Nacional do Catimbau, Olho D’água do Pico; alt. 763 m; 08°33′27.6″ S, 37°11′42.2″ W; 15.III.2021; J.M.S. Rodrigues & R. Jordão leg.; 1 ♂, 2 ♀, CEIOC.

Distribution. Brazil (RN, PE*, BA).

**Figure 10:**
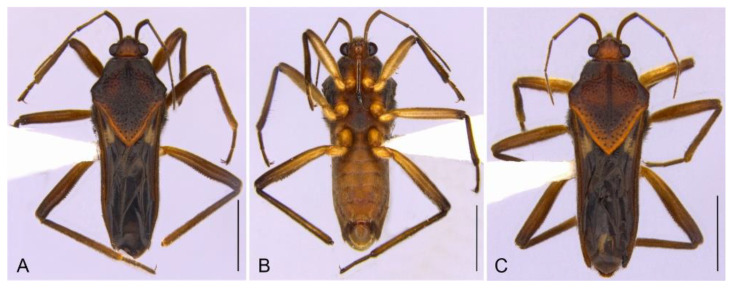
*Paravelia nieseri* from Piauí state, macropterous males, habitus. (**A**,**C**). Dorsal view. (**B**). Ventral view. Scale bars: 2 mm.

**Figure 11:**
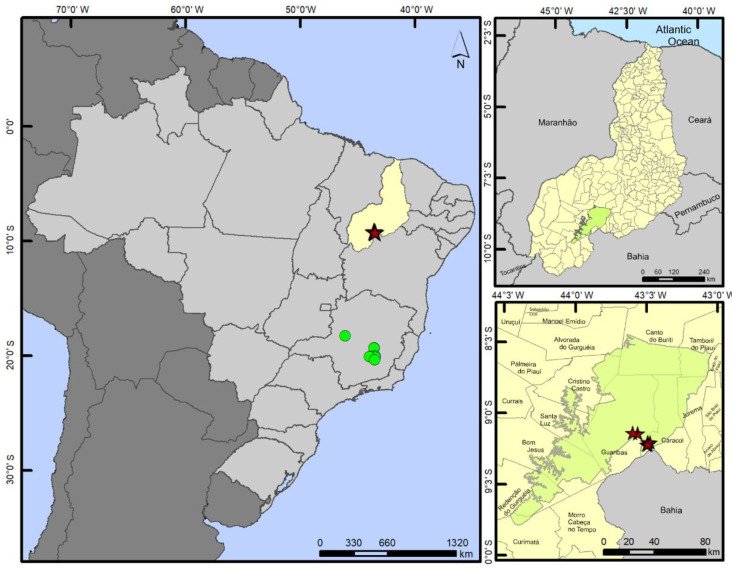
Geographic distribution of *P. nieseri*. Green circles show the previous records in Minas Gerais state. Purple stars show the new records from Serra das Confusões National Park (highlighted in green), Piauí state (highlighted in yellow).

**Figure 12:**
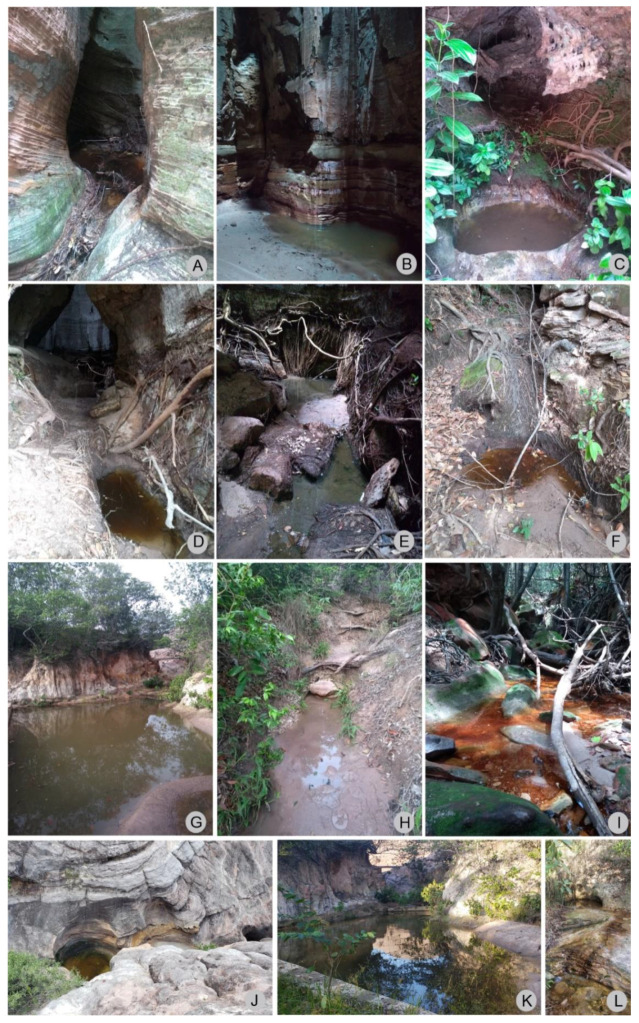
Photographs of the localities where *P. nieseri* was collected in Serra das Confusões National Park, Piauí state, Brazil. (**A**–**C**,**G**–**L**). Caracol municipality. (**D**–**F**). Guaribas municipality. (**A**–**C**). Puddles in the Catedral; 09°08′15.6″ S, 43°35′50.6″ W. (**D**–**F**). Puddles; 09°08′24.9″ S, 43°33′41.0″ W. (**G**). Swamp, between the lookout *Janela do Sertão* and the cemetery; 09°13′09.1″ S, 43°29′24.8″ W. (**H**). Stream, between the lookout *Janela do Sertão* and the cemetery; 09°12′07.5″ S, 43°29′24.6″ W. (**I**). Stream; 09°13′16.6″ S, 43°29′43.5″ W. (**J**). Puddle in the *Cores da Caatinga* trail; 09°12′17.7″ S, 43°28′21.9″ W. (**K**,**L**). Puddles beside the road, near the cemetery, 09°13′06.5″ S, 43°29′25.3″ W.

***Paravelia nieseri*Moreira & Barbosa, 2012** (Figure 10, Figure 11 and Figure 12)

Material examined. BRAZIL–Piauí • Caracol, Parque Nacional da Serra das Confusões, entre o mirante Janela do Sertão e o cemitério; alt. 566 m; 09°13′09.1″ S, 43°29′24.8″ W; 10.XII.2018; J.M.S. Rodrigues & O.M. Magalhães leg.; 1 ♂, CEIOC • same data except: 09°12′07.5″ S, 43°29′24.6″ W; 2 ♂, 3 ♀, CEIOC • Guaribas, Parque Nacional da Serra das Confusões; alt. 539 m; 09°08′24.9″ S, 43°33′41.0″ W; 11.XII.2018; J.M.S. Rodrigues & O.M. Magalhães leg.; 4 ♂, 2 ♀, CEIOC • Caracol, Parque Nacional da Serra das Confusões, poças na Catedral; alt. 524 m; 09°08′15.6″ S, 43°35′50.6″ W; 11.XII.2018; J.M.S. Rodrigues & O.M. Magalhães leg.; 19 ♂, 13 ♀, CEIOC • Caracol, Parque Nacional da Serra das Confusões, riacho próximo aos olhos d’água; alt. 551 m; 09°13′16.6″ S, 43°29′43.5″ W; 12.XII.2018; J.M.S. Rodrigues & O.M. Magalhães leg.; 1 ♂, CEIOC • Caracol, Parque Nacional da Serra das Confusões, Cores da Caatinga, poça próxima ao rio seco; alt. 547 m; 09°12′17.7″ S, 43°28′21.9″ W; 13.II.2020; J.M.S. Rodrigues & I.R.S. Cordeiro leg.; 8 ♂, 2 ♀, CEIOC • Caracol, Parque Nacional da Serra das Confusões, poças ao lado da estrada, próximas ao cemitério; alt. 565 m; 09°13′06.5″ S, 43°29′25.3″ W; 13.II.2020; J.M.S. Rodrigues & I.R.S. Cordeiro leg.; 1 ♂, 2 ♀, CEIOC.

Distribution. Brazil (PI*, MG).

### 3.3. Key to the Species of Paravelia Recorded from Brazil

(Modified from [1,3]; distribution in Brazilian states presented next to each species)

1. Body length less than 3.40 mm … 2
  - Body length equal to or more than 3.40 mm … 3
2. Head, pronotum, legs, and abdomen covered by small black denticles; anterior lobe of pronotum with a pair of pruinose areas laterally [1] (p. 34, Figure 18C,D) … *P. splendoris* (Drake & Harris, 1933) (PA, MT, GO, MG, ES)
  - Body and legs without black denticles, covered by very long setae; anterior lobe of pronotum without pruinosity [1] (p. 25, Figure 14C,D) … *P. capixaba* Moreira, Nessimian & Rúdio, 2010 (AM, PA, MG, ES, RS)
3. Forewings with bubble-like structures on basal region near humeral angles [1] (p. 25, Figure 14A) … *P. bullialata* Polhemus & Polhemus, 1984 (AM, PA, RO)
  - Forewings without bubble-like structures … 4
4. Venter of abdomen covered by rounded or oval punctations (as in Rodrigues et al. [1] (p. 12, Figure 6D)) … 5
  - Venter of abdomen not covered by punctations … 9
5. Male with pronotum distinctly widened anteriorly [1] (p. 27, Figure 15B); female without widened pronotum; body length equal to or more than 5.30 mm … *P. dilatata* Polhemus & Polhemus, 1984 (AM, PA)
  - Pronotum never widened anteriorly; body length less than 5.30 mm … 6
6. Basal macula of forewing distinctly narrowed on distal half; a small rounded white macula present on side of basal macula [1] (p. 31, Figure 17A) … *P. foveata* Polhemus & Polhemus, 1984 (RR, AM)
  - Basal macula of forewing not distinctly narrowed on distal half; additional rounded maculae absent from base of wing … 7
7. Distance between basal and apical maculae of forewings smaller than length of basal macula; apical macula slightly constricted medially [12] (p. 131, Figure 1) … *P. hungerfordi* (Drake & Harris, 1933) (MT)
  - Distance between basal and apical maculae of forewings distinctly greater than length of basal macula; apical macula distinctly constricted medially (as in Rodrigues et al. [1] (p. 6, Figure 1A)) … 8
8. Apical macula of forewing strongly constricted medially, sometimes discontinued on median region (Figure 6A; [1] (p. 9, Figure 4A,B)); posterior margin of male abdominal sternum VII with two small lobes [1] (p. 12, Figure 6D); male proctiger with a pair of bilobed projections [1] (p. 12, Figure 6E,F) … *P. bilobata* Rodrigues, Moreira, Nieser, Chen & Melo, 2014 (CE, MT)
  - Apical macula of forewing less constricted medially [1] (p. 6, Figure 1A); posterior margin of male abdominal sternum VII without lobes, almost straight [1] (p. 6, Figure 1B); projections of male proctiger not bilobed [1] (p. 7, Figure 3A,B) … *P. amapaensis* Rodrigues, Moreira, Nieser, Chen & Melo, 2014 (AP)
9. Forewing with an elongated macula basally and a pair of laterally placed rounded maculae apically; all maculae whitish (as in Rodrigues et al. [1] (p. 15, Figure 14B)) … 10
  - Forewing maculae not as above … 11
10. Body length 3.60–4.20 mm; legs brown, lighter towards base, unarmed; forewing with basal macula long, starting close to humeral angle of pronotum … *P. capillata* (Drake & Harris, 1933) (SE, MT)
  - Body length 4.55–4.95 mm; legs annulate, with black spinules on femora and tibiae; forewing with basal macula short, starting after apex of pronotum … *P. cognata* (Drake & Harris, 1933) (MA, MT)
11. Forewing with basal and apical maculae small, of similar size, and rounded [1] (p. 16, Figure 8A) … *P. micromaculata* Rodrigues, Moreira, Nieser, Chen & Melo, 2014 (PA, MA)
  - Forewing with basal and apical maculae of different sizes and/or shapes … 12
12. Pronotal humeral angle with or without spinose projection [4] (p. 186, Figures 9 and 10); forewing with a subtriangular white basal macula along costal margin, at apex a central, oval, whitish area and a pair of irregular maculae on margins [1] (p. 36, Figure 19B) … *P. spinifera* Polhemus & Polhemus, 1984 (PA, MA)
  - Pronotal humeral angle never with spinose projection; forewing maculae not as above … 13
13. Head, thorax, and abdominal laterotergites covered by small black denticles; pronotum orange or yellowish-orange; forewing with an additional white stripe in front of the basal macula; one macula present on cubital vein and a whitish line surrounding the wing; at apex, another white macula (as in [1] (pp. 16, 34, Figures 10A and 18B)) … 14
  - Black denticles, if covering body, not distributed as above; pronotum with variable coloration; forewing at most with basal and apical maculae … 16
14. General body color yellowish-orange; body length 5.00 mm; male abdominal sternum VII with a pair of distinct projections [13] (p. 27, Figure 9) … *P. confusa* (Hungerford, 1930) (AM, PA)
  - General body color orange to orange-brown; body length 3.80–4.25 mm; male abdominal sternum VII without projections (as in [1] (p. 18, Figure 10C)) … 15
15. Antennomere IV whitish, with small brown areas on base and apex [1] (p. 34, Figure 18B); male proctiger with a distinct horn-like expansion on basal region and small black denticles on apex [1] (p. 37, Figure 20D); posterior angle of last abdominal laterotergite of female not developed … *P. rotundanotata* (Hungerford, 1930) (MT, MS, MG)
  - Antennomere IV entirely orange-brown [1] (p. 18, Figure 10A); male proctiger only with a basal elevation, without small black denticles on apex [1] (p. 21, Figure 12B); posterior angle of last abdominal laterotergite of female developed, acute [1] (p. 21, Figure 12D) … *P. ornata* Rodrigues, Moreira, Nieser, Chen & Melo, 2014 (AM)
16. Forewing with basal macula distinctly yellow (Figures 1A and 2A; [1] (pp. 20, 22, Figures 11A and 13A,B)) … 17
  - Forewing with basal macula white or yellowish-white … 20
17. Male abdominal sternum VII with a pair of large projections ([13] (p. 27, Figure 11); [6] (p. 4, Figure 2D)) … 18
  - Male abdominal sternum VII without projections or lobes … 19
18. Thorax and abdomen dark-brown to black; basal macula of forewing starting from humeral angle; apical macula of forewings, when present, narrow and elongated [1] (p. 22, Figure 13A,B); hind femur without spines … *P. basalis* (Spinola, 1837) (MG, ES, RJ, SP)
  - Thorax dark-brown, abdomen ventrally light-brown to brown; basal macula of forewing starting close to humeral angle; apical macula of forewings rounded [1] (p. 20, Figure 11A); hind femur with spines ([1] (p. 21, Figure 12F); [6] (p. 6, Figure 3J)) … *P. polhemusi* Rodrigues, Moreira, Nieser, Chen & Melo, 2014 (PA, MA, MT)
19. Basal macula of forewing starting from humeri and surpassing posterior margin of pronotum; apical macula of forewing elongated-oval, almost reaching apex of wing [3], p. 644, Figure 9); male proctiger with a conical process at base [3] (p. 646, Figure 21) … *P. luderwaldti* Rodrigues & Moreira, 2016 (SP)
  - Basal macula of forewing not reaching humeri or posterior margin of pronotum; apical macula of forewing rounded to oval, located far from apex of wing (Figures 1A and 2A); male proctiger without conical process at base (Figure 3C) … *P. luisi* Rodrigues & Moreira, sp. nov. (PE)
20. Body with small black denticles, which can be restricted to male abdominal segment VIII (in *P. cunhai*; [4] (p. 185, Figures 5 and 6)) or more widespread … 21
  - Small black denticles completely absent from body … 30
21. Posterior angle of pronotum ending in a robust, finger-like, upward directed process (Figure 8B; [3] (p. 644, Figure 6)); small black denticles on ventral region of head, prosternum, abdominal sterna III–IV, and male abdominal segment VIII; male abdominal sternum VII with a pair of nodules on posterior region (Figure 8C; [3] (p. 656, Figure 31) … *P. digitata* Rodrigues & Moreira, 2016 (RN, PE, BA)
  - Posterior angle of pronotum without an upward directed process; small black denticles with different disposition; male abdominal sternum VII without nodules on posterior region … 22
22. Posterior angle of pronotum acuminated (as in [1] (p. 31, Figure 17D)); male abdominal sternum VII with a pair of acute projections [14] (p. 62, Figure 13) … 23
  - Posterior angle of pronotum rounded (as in [1] (p. 31, Figure 17B)) to slightly acute (as in [1] (p. 22, Figure 13C)), not acuminated; male abdominal sternum VII without projections … 24
23. Pronotum orange, with anterior lobe, humeral angles, and wide median stripe brownish; base of paramere with dorsal surface slightly widened [15] (p. 167, Figure 2) … *P. truxali* Polhemus & Polhemus, 1985 (GO)
  - Pronotum dark-brown to black, with margins of posterior lobe orange (Figure 10A,C; [1] (p. 31, Figure 17D)); paramere with a small basal notch on dorsal surface [1] (p. 37, Figure 20A) … *P. nieseri* Moreira & Barbosa, 2012 (PI, MG)
24. Small black denticles present on abdominal laterotergites; body length 3.54–4.00 mm … 25
  - Small black denticles absent from abdominal laterotergites; body length 4.42–5.52 mm … 27
25. Body length 3.54 mm; abdominal segment VIII of male without small black denticles; paramere curved mesally, becoming darker and narrower towards apex … *P. nexa* (Drake & Harris, 1933) (MA)
  - Body length 4.00 mm; abdominal segment VIII of male with small black denticles laterally; paramere not as above … 26
26. Paramere curved, constricted near base and after middle, with wide apex … *P. kahli* (Drake & Harris, 1933) (MT)
  - Paramere with apex strongly curved and acute … *P. parilis* (Drake & Harris, 1933) (MT)
27. Anterior lobe of pronotum laterally with a pair of silvery pubescent areas [1] (p. 7, Figure 2A,B); abdominal sterna covered by small black denticles … *P. bahiana* Rodrigues, Moreira, Nieser, Chen & Melo, 2014 (BA)
  - Anterior lobe of pronotum without silvery pubescence [1] (pp. 15, 31, Figures 7A and 17B); abdominal sterna with or without small black denticles … 28
28. Body length 5.52 mm; forewing with a white, tear-like, basal macula starting from humeral angle and surpassing posterior margin of pronotum [1] (p. 15, Figure 7A) … *P. lacrymosa* Rodrigues, Moreira, Nieser, Chen & Melo, 2014 (MG)
  - Body length 4.42–5.15 mm; forewing with a white, oval, basal macula not reaching humeral angle and ending adjacent to posterior margin of pronotum or slightly beyond [1] (p. 31, Figure 17B); [4] (p. 185, Figure 1) … 29
29. Grasping comb occupying 1/5 of fore tibial length; hind femur with a row of spines [4] (p. 185, Figure 4); abdominal sterna II–VI without black denticles [4] (p. 185, Figure 2) … *P. cunhai* Rodrigues & Moreira, 2016 (PA)
  - Grasping comb occupying 1/3 of fore tibial length; hind femur without spines; abdominal sterna II–VI with black denticles … *P. lanemeloi* Moreira & Barbosa, 2012 (MG)
30. Body length equal to or more than 4.90 mm; non-bromelicolous species … 31
  - Body length less than 4.90 mm; bromelicolous species … 34
31. Posterior angle of pronotum terminating in a digitiform process ([1] (p. 8, Figure 3F); [4] (p. 186, Figure 8)); male abdominal sternum VII without projections or lobes on posterior margin … 32
  - Posterior angle of pronotum rounded (as in [1] (p. 36, Figure 19A,C); male abdominal sternum VII with a pair of projections [1] (p. 36, Figure 19D) or with two small lobes medially on posterior margin [13] (p. 27, Figure 4) … 33
32. Body length equal to or less than 6.00 mm; forewing with apical macula projected laterally in the distal region [4] (p. 186, Figure 8); paramere slightly narrowed on base, without dorsal notch [16] (p. 504, Figure 5) … *P. juruana* Polhemus & Polhemus, 1984 (AM)
  - Body length more than 6.00 mm; forewing with apical macula evenly ovate, not projected laterally [1] (p. 22, Figure 13C); paramere slightly curved, with a dorsal notch near base [17] (p. 363, Figure 6) … *P. biae* Spangler, 1989 (PA, RO)
33. Femora yellowish on basal half, remaining of legs brownish [1] (p. 36, Figure 19C); male with a pair of distinct projections on posterior margin of abdominal sternum VII [1] (p. 36, Figure 19D); body length 5.80–6.00 mm … *P. williamsi* (Hungerford, 1930) (AM)
  - Legs dark-brown [1] (p. 36, Figure 19A); posterior margin of male abdominal sternum VII medially extended, forming two small lobes on sides of a central concavity [13] (p. 27, Figure 4); body length 5.03 mm … *P. platensis* (Berg, 1883) (SP)
34. Anterior lobe of pronotum without pubescence laterally [3] (p. 655, Figure 29) … *P. itatiayana* (Drake, 1951) (RJ, SP)
  - Anterior lobe of pronotum with a pair of lateral, white, pubescent areas [3] (p. 643, Figures 1, 3–5, 27 and 30) (some specimens of *P. recens* may have the pubescence irregular and not so evident; see [1] (p. 34, Figure 18A) … 35
35. Pubescence of anterior lobe of pronotum narrow, elongate, and slightly curved laterally [3] (p. 655, Figure 27); distance between basal and apical maculae of forewing approximately one-third the length of the apical macula [3] (p. 655, Figure 27) … *P. gabrielae* Moreira & Barbosa, 2011 (ES, SP)
  - Pubescence of anterior lobe of pronotum subtriangular [3] (p. 644, Figures 1 and 3–5), rectangular [3] (p. 655, Figure 30), or irregular [3] (p. 658, Figure 48); distance between basal and apical maculae of forewing equal to or longer than the length of the apical macula [3] (pp. 644, 655, 658, Figures 1, 3–5, 30 and 48) … 36
36. Body black [3] (p. 655, Figure 30); pubescence of anterior lobe of pronotum rectangular [3] (p. 655, Figure 30); male proctiger with two or three elevations on dorsal surface [3] (p. 656, Figure 32); paramere as in [3] (p. 656, Figure 32) … *P. manausana* Polhemus & Polhemus, 1984 (AM)
  - Body brown, reddish-brown, or orange-brown [3] (pp. 644, 658, Figures 1, 3–5 and 48); pubescence of anterior lobe of pronotum subtriangular [3] (p. 644, Figures 1 and 3–5) or irregular [3] (p. 658, Figure 48); male proctiger with at most one dorsal elevation plus a small acute process [3] (pp. 646, 656, Figures 12, 15 and 41); paramere not as above … 37
37. Apical macula of forewing usually rounded or oval, shorter than basal macula [3] (p. 658, Figure 48); male proctiger without elevation or acute process anteriorly [3] (p. 656, Figure 41) … *P. recens* (Drake & Harris, 1935) (AM, PA, ES)
  - Apical macula of forewing narrow and elongate, as long as or longer than basal macula [3] (p. 644, Figures 1 and 3–5); base of male proctiger with a weak elevation [3] (p. 646, Figure 12) or a weak elevation plus small acute process [3] (p. 646, Figure 15) … 38
38. Male proctiger only with a weak elevation at base [3] (p. 646, Figure 12); paramere as in [3] (p. 646, Figure 12) … *P. bachmanni* Rodrigues & Moreira, 2016 (SP, SC)
  - Male proctiger with a weak elevation plus a small acute process at base [3] (p. 646, Figure 15); paramere as in [3] (p. 646, Figure 15) … *P. bromelicola* Rodrigues & Moreira, 2016 (SP, SC)

## 4. Discussion

Our collections at conservation areas in three of the eight Brazilian states with no previous occurrences of *Paravelia* (Ceará, Pernambuco and Piauí) [10] resulted in the description of a new species, *P. luisi* **sp. nov.**, and new records of three species: *P. bilobata, P. digitata*, and *P. nieseri*. This increases the number of valid *Paravelia* species to 50 and the number of species recorded in Brazil to 39. Our report of *P. bilobata* constitutes the first record of the genus from Ceará (Aiuaba Ecological Station) and extends the known distribution of the species by about 1600 km to the northeast [1]. *Paravelia digitata* and *P. luisi*, **sp. nov.**, in turn, represent the first records of the genus from Pernambuco (Catimbau National Park). The former species had been recorded in Rio Grande do Norte [3] (about 290 km to the north of our sampling site) and in Bahia [3,18] (the nearest record to ours was at about 600 km to the southwest). Likewise, *P. nieseri* is recorded for the first time in Piauí (Serra das Confusões National Park). It was previously known in Minas Gerais [1,3,14], with the nearest record to ours about 1000 km to the south.

Finally, after the latest revision of *Paravelia* by [1], there have been several changes to the composition of the genus. For example, [3] described *P. bachmanni, P. bromelicola, P. luederwaldti,* and *P. digitata*; [4] described *P. cunhai* and synonymized *P. cupariana* with *P. spinifera*; and [8] transferred *P. bipunctata* Rodrigues, Moreira, Nieser, Chen & Melo, 2014, *P. conata* (Hungerford, 1929), and *P. taipiensis* (Cheesman, 1926) (= *P. virtutis* Drake & Harris, 1935) to the genus *Callivelia*. Therefore, we presented an updated key to the species of *Paravelia* occurring in Brazil, which will aid in the proper identification of specimens of the genus.

## Data Availability

The data presented in this study are available in the article.
